# Experimental optimization of the energy for breast-CT with synchrotron radiation

**DOI:** 10.1038/s41598-020-74607-7

**Published:** 2020-10-15

**Authors:** Piernicola Oliva, Vittorio Di Trapani, Fulvia Arfelli, Luca Brombal, Sandro Donato, Bruno Golosio, Renata Longo, Giovanni Mettivier, Luigi Rigon, Angelo Taibi, Giuliana Tromba, Fabrizio Zanconati, Pasquale Delogu

**Affiliations:** 1 Dipartimento Di Chimica E Farmacia, Università Di Sassari, Sassari, Italy; 2 I.N.F.N. Sezione Di Cagliari, Cagliari, Italy; 3 Dipartimento Di Scienze Fisiche, Della Terra E Dell’Ambiente, Università Di Siena, Siena, Italy; 4 I.N.F.N. Sezione Di Pisa, Pisa, Italy; 5 Dipartimento Di Fisica, Università Di Trieste, Trieste, Italy; 6 I.N.F.N. Sezione Di Trieste, Trieste, Italy; 7 Dipartimento Di Fisica, Università Della Calabria, Cosenza, Italy; 8 I.N.F.N. Laboratori Nazionali Di Frascati, Frascati, Italy; 9 Elettra-Sincrotrone Trieste SCpA, Basovizza, Italy; 10 Dipartimento Di Fisica, Università Di Cagliari, Cagliari, Italy; 11 Dipartimento Di Fisica, Università Di Napoli Federico II, Napoli, Italy; 12 I.N.F.N. Sezione Di Napoli, Napoli, Italy; 13 Dipartimento Di Fisica E Scienze Della Terra, Università Di Ferrara, Ferrara, Italy; 14 I.N.F.N. Sezione Di Ferrara, Ferrara, Italy; 15 Dipartimento Di Scienze Mediche Chirurgiche E Della Salute, Università Di Trieste, Trieste, Italy

**Keywords:** Biological physics, Imaging techniques

## Abstract

Breast Computed Tomography (bCT) is a three-dimensional imaging technique that is raising interest among radiologists as a viable alternative to mammographic planar imaging. In X-rays imaging it would be desirable to maximize the capability of discriminating different tissues, described by the Contrast to Noise Ratio (CNR), while minimizing the dose (i.e. the radiological risk). Both dose and CNR are functions of the X-ray energy. This work aims at experimentally investigating the optimal energy that, at fixed dose, maximizes the CNR between glandular and adipose tissues. Acquisitions of both tissue-equivalent phantoms and actual breast specimens have been performed with the bCT system implemented within the Syrma-3D collaboration at the Syrmep beamline of the Elettra synchrotron (Trieste). The experimental data have been also compared with analytical simulations and the results are in agreement. The CNR is maximized at energies around 26–28 keV. These results are in line with the outcomes of a previously presented simulation study which determined an optimal energy of 28 keV for a large set of breast phantoms with different diameters and glandular fractions. Finally, a study on photon starvation has been carried out to investigate how far the dose can be reduced still having suitable images for diagnostics.

## Introduction

Early detection of breast cancer increases the effectiveness of treatments and reduces the mortality of 20%^1^. Breast-cancer screenings for women older than 40 years are nowadays performed as routine in industrialized countries, especially through dual view mammography^2–4^. Being a 2D imaging technique, the diagnostic power of mammography is limited by the superimposition of anatomical structures which can hinder the detection of clinically relevant features. This limitation leads to a not-negligible number of false-positives and false-negatives^1,5–8^.

Among the alternative approaches to breast imaging, X-ray breast Computed Tomography (bCT) is showing promising results as non-invasive tool for detecting breast cancer, thanks to a reduction of the risk for misleading diagnosis. In particular, bCT is a 3D imaging technique that totally removes overlaps and increases the visibility among breast tissues at the cost of an increased radiation dose and a reduction of spatial resolution^9–12^. At present, only two dedicated bCT systems are commercially available^13,14^. These systems allow for conventional absorption tomography of the breast and they make use of X-ray tubes which emit polychromatic radiation.

If compared to polychromatic radiation, the use of monochromatic radiation allows for a dose reduction while preserving the image quality^15–17^. Synchrotron sources provide access to monochromatic X-rays. Moreover, thanks to the high spatial coherence of synchrotron radiation, the propagation-based phase-contrast (PhC) imaging technique can be implemented. Images acquired in the propagation-based configuration, which show the so-called edge-enhancement effect arising at the interfaces between different materials, are usually further processed with a phase-retrieval (PhR) algorithm, as the one proposed by Paganin et al.^18^, whose final effect is to increase the signal-to-noise ratio, while maintaining the same contrast and spatial resolution of conventional absorption imaging^19–22^. Specifically, considering low-dose bCT images, it has been demonstrated that the PhR filter is required to provide a good visibility of breast tissues (e.g., adipose, fibroglandular, skin) which feature poor attenuation contrast^23^.

In this context, an experimental setup for phase-contrast bCT has been implemented at the SYRMEP beamline of Elettra synchrotron facility (Trieste, Italy) within the framework of the Syrma-3D (Synchrotron Radiation Mammography-3D) collaboration. Syrma-3D uses Pixirad-8 detection system, an X-ray photon counting detector mounting a 650 μm thick CdTe sensor with a pixel pitch of 60 μm^24,25^. The collaboration demonstrated that, by combining the advantages of monochromatic radiation with PhC techniques and a highly efficient photon counting detection system, the imaging of breast samples is possible at doses and acquisition-times compatible with the clinical practice^26–28^.

Promising results have been recently obtained also at the Australian Synchrotron, where surgically excised breast mastectomy specimens have been scanned with PhC bCT, achieving high radiological image quality at low dose^29,30^.

Due to the high radiosensitivity of the glandular component of breast tissues, particular attention has to be paid to the dose delivered to the irradiated tissues. For breast imaging, the reference parameter for dose calculation is the mean glandular dose (MGD), which is the total energy deposited in the glandular tissues divided by the total mass of the breast^31^. Since a direct measure of the MGD is not possible, this parameter is usually evaluated through Monte Carlo simulations^11,32–36^. These simulations consider the X-ray spectrum, the geometry, the glandularity and the size of the breast to be imaged to calculate the normalized glandular dose (*DgN*). In these models, the MGD is defined as the product between the *DgN* with the air kerma *K* at the entrance surface of the breast (i.e. $MGD=K\cdot DgN$ ). In this paper, we refer to (Mettivier et al.^36^) for MGD evaluations.

The optimization of an X-ray imaging system requires to find the best trade-off between the dose delivered to the patient and the image quality. In assessing image quality, the discriminating power between two biological tissues of different elemental composition, described by the contrast to noise ratio (CNR), plays a crucial role. Since at fixed fluence both dose and CNR are decreasing functions of the energy, the choice of the energy for bCT has a crucial role to achieve the best CNR. In the framework of SYRMA-3D collaboration, the optimization of the energy for conventional absorption bCT has been calculated^37^. In (Delogu et al.^37^), the authors derived an analytical formula for CNR and developed an analytical simulator to produce synthetic bCT images of an idealized cylindrical breast sample. A systematic study to find the optimal energy for bCT has been performed using the CNR as the figure of merit. The authors found out that, considering breasts with different diameters and glandularities, a good compromise between CNR and MGD is achieved with monochromatic photons around *28* keV for a large range of breast sizes and glandularities. Moreover, the authors showed that even if the delivered dose does not influence the energy dependence of the analytical CNR, photon starvation artifacts appear when the number of detected photons is too low, resulting in images not suitable for diagnostic purposes. This study was based on theoretical considerations and did not take into account PhC effects.

A first aim of this paper is to experimentally investigate the energy dependence of CNR for a set of tissue-equivalent phantoms and breast specimens, also considering the effects of PhR algorithm on the optimal energy in bCT.

A second aim is the validation of the analytical simulator for bCT developed in our previous work^37^ through a comparison with experimental data. For this purpose, well-characterized phantoms and actual breast samples were both imaged and simulated. The study of the behavior of the CNR against the energy at fixed dose has been conducted by comparing the measurement from experimental data and simulations. Since the experimental images of breast samples are reconstructed using a PhR algorithm, in order to obtain a better image quality at acceptable dose levels, the PhR has been implemented in the simulation process.

Finally, the effects of the photon starvation on the optimal energy have been experimentally investigated on different breast tissues.

## Materials and methods

### Experimental setup

Experiments were performed at the SYRMEP beamline of the Elettra (Trieste, Italy) facility^38^. The only optical element of the beamline is a Si(111) double-crystal monochromator, which provides tunable monoenergetic X-rays in the range 8–40 keV, with a resolution of 0.1%. At the sample position, 30 m away from the bending magnet source, the fan beam measures about 220 mm horizontally and 3.5 mm vertically (Gaussian shape, FWHM). The patient support developed for the mammography program^39^ was used to hold the phantoms and the surgical specimens during image acquisition. In order to detect phase-contrast effects, the detector was placed 1.6 m away from the sample. A custom dosimetric system, based on two custom-made high-precision ionization chambers placed approximately 3 m upstream from the sample, was used to define the exposure parameters. The ionization chambers measure the entrance radiation dose in terms of absolute air kerma and were calibrated by the Department of Ionizing Radiation Metrology of the Italian National Agency for New Technologies, Energy and Environment (ENEA)^40,41^. This system is used to calculate the MGD delivered to the sample throughout the tomographic acquisition.

The imaging detector, Pixirad-8, is a CdTe direct-conversion high-efficiency photon-counting device with a linear response up to 2 × 10^5^counts pixel^−1^ s^−1^^42^. The large active area of 246 mm × 24.8 mm is obtained by tiling eight modules, 30.7 mm × 24.8 mm each. The detector features 4096 × 476 pixels, arranged on a honeycomb matrix with a 60 µm pitch. Each pixel is associated with two independent 15-bit counters, which can be used to obtain a negligible dead-time between frames (dead-time-free mode). In practice, both counters thresholds are set to the same value and one counter is filled while the other is being read. This modality has been used in the present study to perform a continuous irradiation of the samples without losing counts or requiring a synchronized shutter. During the acquisitions with this modality, the detector threshold has been set to 3 keV to safely remove the electronic noise while preserving the detection efficiency^43^. Further details on the detector performances (e.g. spatial resolution, noise, operation modes) are reported by (Delogu et al*.*^24^).

The projection images undergo an ad-hoc developed pre-processing procedure tailored on the Pixirad-8 characteristics. The aim of the procedure is to remove artifacts in the image, mainly due to non-uniform response of the detector. The procedure has been thoroughly discussed by (Brombal et al.^44^).

For the tomographic scans, 1200 projections were acquired over 180 degrees in a total exposure time of 40 s, corresponding to a continuous rotation speed of 4.5 degrees/s and a 30 Hz detector frame rate.

### Phantoms

We used two phantoms to investigate the energy dependence of CNR and compare the experimental results with those obtained from the analytical simulation. These phantoms are meant to provide an experimental test of the analytical simulation in controlled conditions. To allow for an easy implementation of simulations, phantoms with cylindrical symmetry have been employed. Homogenous materials were used in order to allow a direct measure of the linear attenuation coefficients and to avoid problems arising from anatomical noise in the calculation of CNR.

- **P1:** a first phantom was a cylinder (7 cm of diameter), made of adipose-equivalent material, containing a detail (cylinder of 1 cm of diameter, in the center of the phantom) made of glandular-equivalent material. The height of the cylinder was 0.5 cm. The maximum diameter available in our lab for the adipose-equivalent tissue was 7 cm. The tissue-equivalent materials were manufactured by CIRS (Norfolk, VA). Images were acquired from 18 to 38 keV, step 2 keV.
- **P2:** a second phantom was a larger cylinder (12 cm of diameter) made of polyethylene, and containing a detail made of glandular-equivalent tissue (cylinder of 1 cm of diameter in the center of the phantom). The height of the cylinder was 0.5 cm. In this phantom polyethylene is used to mimic the adipose tissue. The glandular-equivalent material was manufactured by CIRS (Norfolk, VA). Images were acquired from 20 to 38 keV, step 2 keV.

Tomographic reconstructions of the two phantoms are reported in Fig. S1 in the Supplementary Materials. These phantoms have been employed to test the agreement between the simulations and the experimental data, thus the equivalence of their composition with actual tissues is not essential. For the tissue-equivalent materials, and also for polyethylene, the linear attenuation coefficients have been experimentally measured as described below.

The regions of interest (ROIs) selected for the calculation of CNR were made of 13,909 pixels for each material.

Images at different energies have been acquired at a constant mean glandular dose of 20 mGy. Even though this dose is higher than the one of 5 mGy suggested for the clinical bCT at Elettra^28^, it allows to better understand the energy dependence of CNR with no consequences in the determination of the optimal energy. In fact, as demonstrated in (Delogu et al.^37^) the energy dependence of CNR is in principle not affected by the chosen value of MGD. However, at low doses, and in particular at low energies, low counts in the detector may generate artifacts in the reconstructed images, which may affect the evaluation of CNR.

### Breast samples

In order to investigate the energy dependence and the position of the maximal CNR in a more realistic environment, we imaged several breast tissues derived from mastectomy specimens. The analyzed surgical samples were fixed in formalin, sealed in a vacuum bag and preserved at room temperature. All the procedures adopted in this work followed the Directive 2004/23/EC of the European Parliament and of the Council of 31 March 2004 on setting standards of quality and safety for the donation, procurement, testing, processing, preservation, storage, and distribution of human tissues. The present study was done in the framework of the operative protocol of the Breast Unit of the Trieste University Hospital (“PDTA Neoplasia mammaria” approved on 11 December 2019 by ASUGI—Azienda Sanitaria Universitaria Giuliana Integrata, Italy). A written informed consent was obtained from all patients prior to their inclusion into the study. The specialist breast center of ASUGI is in compliance with the standard of EUSOMA guidelines (certificate No. 1027/01).

- **T1:** the actual sample is included in a small cylinder (diameter 3.2 cm), containing well-separated adipose and glandular tissues. The cylinder is centered in a polyethylene cylinder, of 12 cm of diameter. Images were acquired from 18 to 38 keV, step 2 keV. The ROIs used for the calculations were made of 85,000 pixels for each material.
- **T2:** mastectomy containing an infiltrating ductal carcinoma, located outside of the volume portion imaged in the present study. The maximum diameter of the sample is 17 cm. Images were acquired at 20, 24, 28 and 38 keV. The ROIs used for the calculations were made of 30,160 pixels for each tissue.
- **T3:** risk reducing mastectomy (no lesion). The maximum diameter is 17 cm. Images were acquired from 20 to 38 keV, step 2 keV. The ROIs used for the calculations were made of 10,944 pixels for each tissue.

As in the phantom study, a high dose (20 mGy) was used in order to obtain a better statistics and to avoid artifacts. As previously outlined, however, the actual value of the MGD does not affect the energy dependence of CNR.

Breast samples T2 and T3 have been already presented in (Piai et al.^45^), as Sample 7 and Sample 4, respectively. Representative slices of the tomographic reconstruction of three samples are reported in Fig. S2 in the Supplementary Materials.

### Linear attenuation coefficients

Linear attenuation coefficients were experimentally measured for tissue-equivalent materials and polyethylene. For the measurements, we used step-wedges made of several slabs of the material (9 steps from 5.05 mm to 45.5 mm for CIRS samples, 6 steps from 5.1 mm to 44.8 mm for polyethylene). The attenuation of each step was evaluated on planar images obtained with the Pixirad-8 detector. The step wedges were 1.6 m away from the detector.

For each monochromatic energy, an image and the corresponding flat-field images were acquired. The flat-field has been averaged over 100 frames. By dividing the image of the step wedge by the flat-field, we directly obtained the image of the transmittance. For each step, the mean value of the counts was measured. The mean transmittance was then fitted using an exponential decay, as a function of material thickness. The procedure was repeated for each energy in the range: 18–38 keV, step 2 keV. Generally, when measuring attenuation coefficient using monochromatic synchrotron radiation, spectral contamination due to the contribution of higher harmonics has to be considered. Nonetheless, in the specific case of the SYRMEP beamline, the bending magnet’s critical energy is quite low (between 4 keV and 5.5 keV) and, for this reason, the higher harmonic contribution is generally rather small. Moreover, the monochromator can be slightly detuned from its maximum reflectivity position to filter out any potential harmonic contamination, in particular at low energies (around 20 keV and below).

For polyethylene, the measured linear attenuation coefficient was also compared with the data from the NIST database (density ρ = 0.93 g/cm^3^).

In terms of linear attenuation coefficients of glandular and adipose tissues, (Piai et al.^45^) showed a substantial agreement of experimental results with previously published data^46,47^. For this reason, the chemical compositions reported in (Hammerstein et al.^31^ and Boone and Chavez^48^) were used for glandular and adipose tissues in actual breast samples, as done in our previous paper^37^. The mass attenuation coefficients, used in the calculations, have been obtained from the NIST database^46^.

### Analytical simulations

In this work we used an analytical simulator for absorption imaging, based on the one described in (Delogu et al.^37^) to generate synthetic two-dimensional projections. The original analytical simulation was modified, in order to use as phantoms two-dimensional pixelated images, with a specific attenuation coefficient assigned to each pixel. The beam was always assumed to be parallel and monochromatic and the scattering was neglected. The object-detector distance was 160 cm, filled with air. The simulation for three-dimensional phantoms was made one slice at a time. The mean glandular dose was set to the same value of the corresponding experimental image. For each simulated CT scan, 1200 projections over 180 degrees have been produced to be coherent with the experimental acquisitions.

The simulated detector is an ideal photon counting system with 100% detection efficiency and square pixels with 60 µm side. In the considered energy range, the assumption of 100% efficiency is reasonable for detectors mounting high-Z sensors. However, the actual detector, Pixirad-8, shows some features that deviate from this ideal model. First of all, a single interacting photon can be counted simultaneously from different adjacent pixels due to the charge sharing effect in the sensor. From the imaging point of view this effect, inducing correlation between pixels that enlarges the PSF, tends to decrease the noise^43^. Secondly, for energies above 26.7 keV, fluorescence photons from Cd (and for Te at energies higher than 31.8 keV) will further enlarge the PSF of the detector inducing again correlation and thus, a slight smoothing of the noise that increases with the energy^24,43,49^.

The small variations mentioned above are expected to impact on the differences between the simulated CNR and the measured one. However, since the energy dependence of these differences is mild (or negligible), the main expected difference is a scale factor between the two CNRs. Since the aim of this work is to investigate the optimization of the detectability of details as a function of energy, the presence of a scale factor does not constitute a limitation.

For the phantoms P1 and P2, the geometry has been directly modeled in the simulations. The linear attenuation coefficients used were the ones experimentally measured, as described in the previous section.

For breast samples, the tomography of reconstructed experimental images was automatically segmented in two types of tissues (adipose and gland) according to the voxel value. For the T1 sample, the images were segmented in three materials (adipose, gland and polyethylene). The resulting masks were used in the analytical simulator to generate the projections. For these breast samples, the simulations have been carried out by using the linear attenuation coefficients of gland and adipose tissues of the breast as described in the previous section.

### Image reconstruction

For both experimental and simulated data, absorption images are directly reconstructed via a GPU-based filtered back-projection (FBP) with a Shepp-Logan filtering^50^.

For phase-retrieved images, the Homogeneous Transport of Intensity (TIE-Hom) Algorithm (Paganin et al.^18^) algorithm was applied to each projection before the actual reconstruction. Phase retrieval can be seen as a two-dimensional filter in the Fourier space *(u, v)* which is written as follows:

1 $$H(u,v)=\frac{1}{1+\pi d\lambda \frac{\delta }{\beta }{\left|w\right|}^{2}}$$

where *λ* is the radiation wavelength, *d* is the propagation distance, $w=\sqrt{{u}^{2}+{v}^{2}}$ is the spatial frequency and δ/β is the ratio between the real decrement and imaginary part of the refraction coefficient and, in Paganin's approach, it is assumed to be constant throughout the sample. For the phase retrieval, the value of δ/β for breast tissue (ICRU-44) was always used. The values of δ and β for each energy were obtained from a publicly available database^51,52^.

A comprehensive discussion on the phase-retrieval filter used for bCT images, encompassing both a theoretical description and experimental results obtained by the SYRMA-3D collaboration, has been given by (Brombal et al.^23^ and Donato et al.^53^).

In principle, the Paganin filter requires a propagation-based phase contrast image in order to correctly retrieve the phase. This is the case for experimental images, while the simulated ones are pure absorption images. However, as can be seen from Eq. (1), the Paganin filter acts essentially as a (bell-shaped) low-pass filter in the frequency domain, whose main effect is a smoothing of the image. Considering typical values for Eq. (1) in our configuration, the width of the Paganin filter in real space is 5–6 pixels. Hence, if we consider a ROI in the image that is more than 10 pixels from any edge between different materials, the effect of the Paganin filter is the same for absorption images and propagation-based phase contrast images. For this reason, the PhR filter has been applied to the simulated absorption images and ROIs have been selected taking into account the previous considerations.

For phantoms P1 and P2, both absorption and phase-retrieved images were reconstructed and compared. For breast tissues only phase-retrieved images were analyzed, since this is the image modality planned for clinical bCT.

### Calculation of CNR

For the calculation of CNR, in a central slice of the experimental images we selected a ROI as large as possible in the glandular (or glandular-equivalent) tissue. A ROI of the same area was then selected in the adipose (or adipose-equivalent) tissue. For each ROI, we verified that it was included in the same tissue/material for at least 10 neighboring slices. Moreover, ROIs were chosen so as to be at least 10 pixels away from any interface between different materials.

The same ROIs were used for the calculation of CNR in absorption and phase retrieved images and for both experimental and simulated images.

CNR was calculated as follows:

2 $$CNR=\frac{{S}_{g}-{S}_{f}}{{\sigma }_{f}}$$

where ${S}_{g}$ is the average gray level in the ROI within the glandular tissue and ${S}_{f}$ and ${\sigma }_{f}$ are the average gray level and the standard deviation in the ROI within the adipose tissue, respectively.

For each image, the calculation was repeated in all the 10 slices and then the average value of the CNR was used and the standard deviation of the mean was reported as statistical error.

### Photon starvation

To better understand the effect of a low number of photons, we investigated the experimental CNR on breast samples at selected energies, as a function of mean glandular dose.

The experimental acquisitions were performed on two samples. For each sample, we selected an energy close to the expected maximal CNR, then we acquired a complete set of projections at different MGDs. The set of acquisitions is reported here:

- Breast sample T1. Energy: 22 keV. Doses: 20, 5, 2, 1, 0.5, 0.25, 0.12 mGy
- Breast sample T3. Energy: 28 keV. Doses: 20, 10, 7.5, 5, 2.5, 1 mGy

For each projection, we calculated the average number of counts *N* in a central area of the image, where the beam is attenuated at most. Then we considered the minimum of *N* on the set of projections used for each reconstruction: *N*_*min*_. On the same projection, we also calculated the percentage of zero-counting pixels in the area used to evaluate *N*_*min*_.

## Results and discussion

### Attenuation coefficients

**Figure 1:**
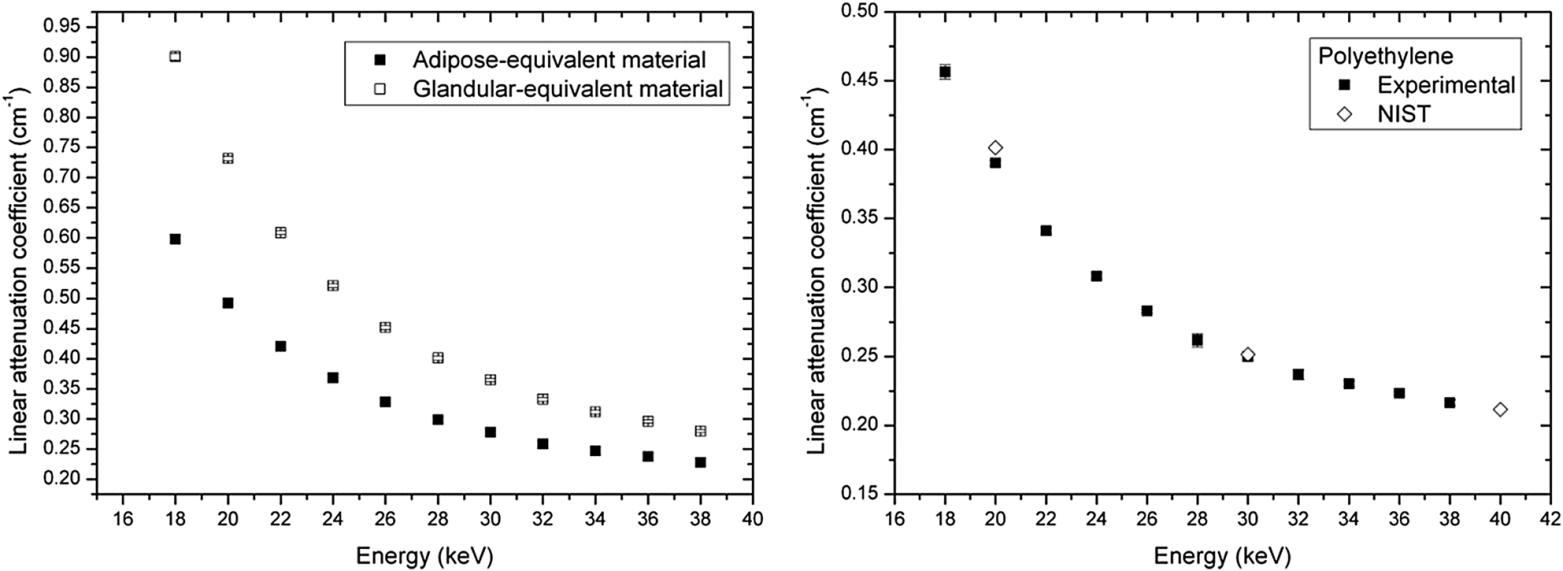
Calculated linear attenuation coefficients. Left: CIRS materials (adipose- and glandular-equivalent, experimental data). Right: Polyethylene (experimental and tabulated data). Standard errors are reported for experimental data, but they are not visible in the figure.

The calculated linear attenuation coefficients for CIRS materials are reported in Fig. 1 (left panel). Standard errors are reported in the plot, but they are not visible since they are smaller than marks. In Fig. 1 (right panel), the measured linear attenuation coefficient (with standard error) of polyethylene is reported. For comparison, tabulated data (NIST database) are also reported.

### Phantoms

**Figure 2:**
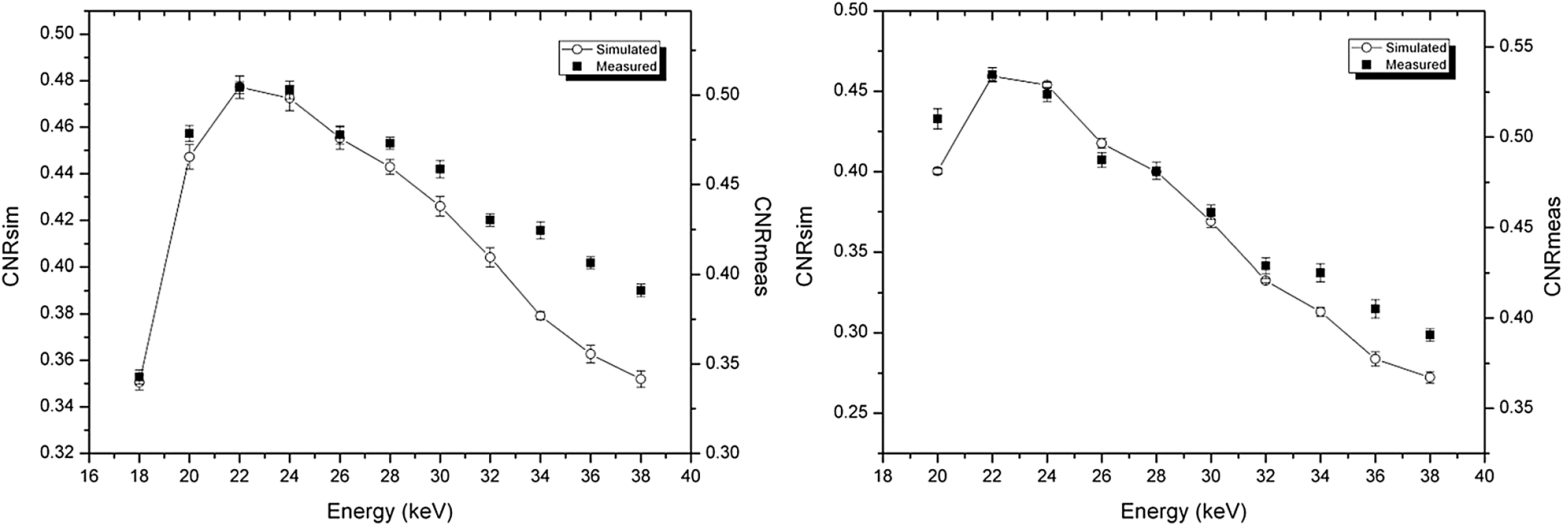
Comparison of CNR for absorption images, calculated from analytical simulation and experimental images. Left: P1 phantom (adipose/glandular, diameter 7 cm). Right: P2 phantom (polyethylene/glandular phantom, diameter 12 cm). The diameter of the glandular detail is 1 cm for both phantoms.

In Fig. 2 the comparisons of absorption CNRs, calculated from the analytical simulation and from the experimental images are reported for both phantoms. The two CNRs are reported with different scales in the same plot. The presence of a scale factor is mainly due to the differences between simulated detector and actual one. As expected, the finite width of the PSF that, due to the charge sharing effect, widens with the increase of the energy mainly determines a difference in scale factors by reducing the noise. While the maxima occur at the same energy for both experimental data and simulated ones, discrepancies in the energy dependence of the CNRs can be observed at energies both higher and lower than the maxima. The differences at low energies are due to the low number of photons reaching the detector, which may lead to pixels with zero counts. This is more pronounced for the phantom with the largest diameter (P2, right panel), where the number of transmitted photons is extremely low at low energies. Thus, the actual gray levels on the reconstructed image depend on how the reconstruction algorithm deals with zero-counting pixels and related artifacts. The differences at high energies may be explained with the further PSF enlargement that occurs at energies above 26.7 keV due to fluorescence of Cd (and Te) induced in the sensor^24,43^. This means that the experimental absorption images are a little smoother at high energy and, therefore, the tail of the curve is less steep.

**Figure 3:**
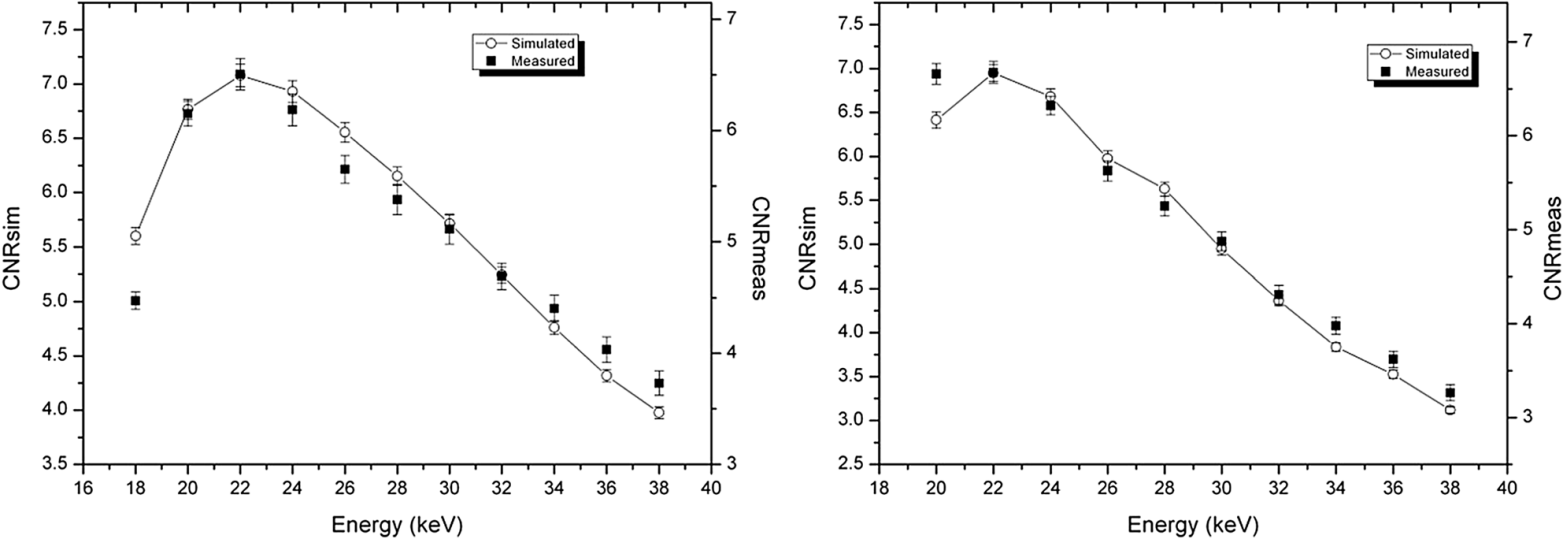
Comparison of CNR for phase-retrieved images, calculated from analytical simulation and experimental images. Left: P1 phantom (adipose/glandular, diameter 7 cm). Right: P2 phantom (polyethylene/glandular phantom, diameter 12 cm). The diameter of the glandular detail is 1 cm for both phantoms.

In Fig. 3, CNRs for phase-retrieved images calculated from experimental images and from simulated data are reported, for the two phantoms. The agreement between simulated data and the experimental ones is fairly good. In particular, the maxima of CNR are at the same energies. The main discrepancies here are at lower energies, because of the zero-counting pixels, as discussed previously. On the other hand, for energies higher than the maximum, the agreement is better for phase-retrieved images than for absorption ones. This is because the smoothing of the Paganin filter (present for both the experimental and simulated images) is dominant with respect to the energy-dependent smoothing due to the widening of the PSF, which characterizes the experimental images.

**Figure 4:**
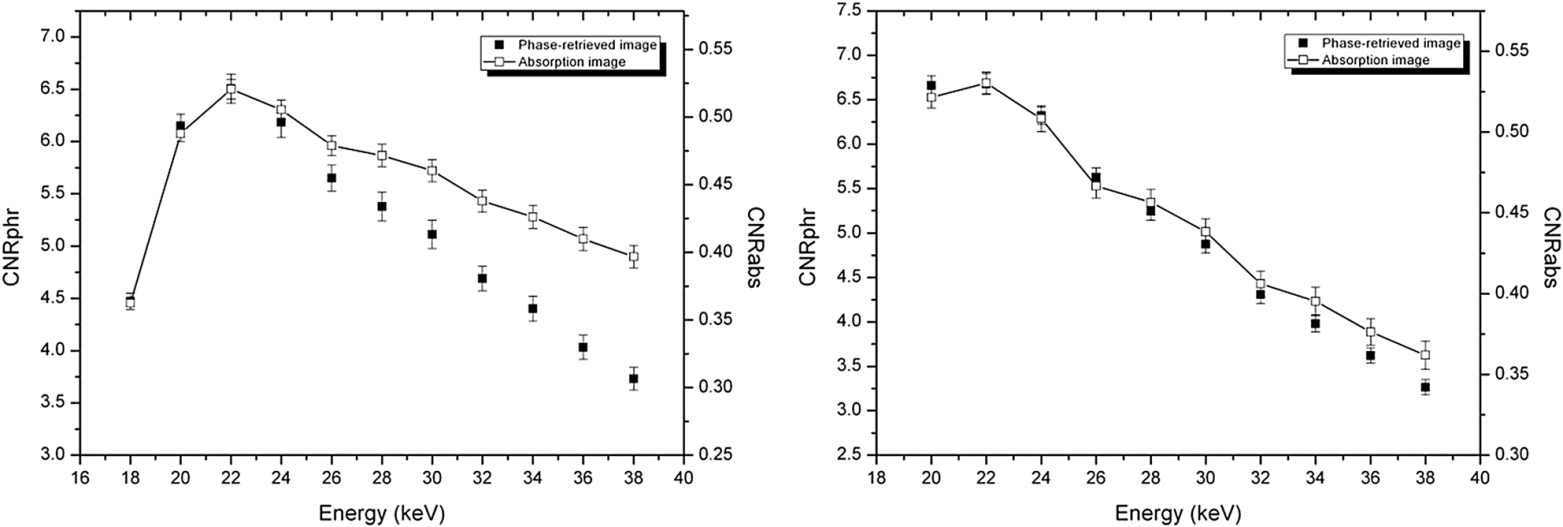
CNR (measured on experimental images) for absorption image and phase-retrieved one, for P1 (left panel) and P2 (right panel) phantoms. CNR for absorption images and for phase-retrieved ones are reported in the same plot with different scales.

In Fig. 4 we present a comparison of CNR (measured on experimental images) as a function of the energy, for both the absorption images and the phase retrieved ones, for the two phantoms P1 (Fig. 4, left panel) and P2 (Fig. 4, right panel). The CNRs for the two image modalities are reported with different scales in the same plot. In this way it is possible to highlight the difference in the values of CNR: for phase-retrieved images, CNR is more than 10 times higher. At the same time, it is possible to appreciate the different energy dependence of the two CNRs.

The maximum of CNR in absorption images is at 22 keV, for both phantoms. The position of the maximum is at the same energy also for phase retrieved images, but in this case the high-energy part of the plot shows a steeper dependence on energy, if compared with absorption images. This effect may be explained by taking into account the energy dependence of the Paganin filter. Often, the energy dependence of the Paganin filter is considered to be weak or absent^54^, since δ is supposed to be proportional to ${\lambda }^{2}$ , while β is supposed to be proportional to ${\lambda }^{3}$ and, therefore, the quantity $\lambda \delta / \beta$ appearing in the filter introduced in Eq. (1) should be a constant. If so, the dependence on energy of CNR would be the same both with and without the application of the phase retrieval. Actually, for the biological tissues and energies under investigation in this study, this is not true. In Fig. S3 in the Supplementary Materials, $\lambda \delta / \beta$ is plotted for breast tissue, as a function of energy. As can be seen, in the case of one-material phase-retrieval, the product $\lambda \delta / \beta$ decreases with increasing energy, i.e. the H filter becomes narrower (in real space), thus producing less smoothing. For this reason, it is reasonable to expect, at high energies, a lower CNR in phase-retrieved images with respect to absorption (rescaled) images, as shown in Fig. 4.

### Breast samples

**Figure 5:**
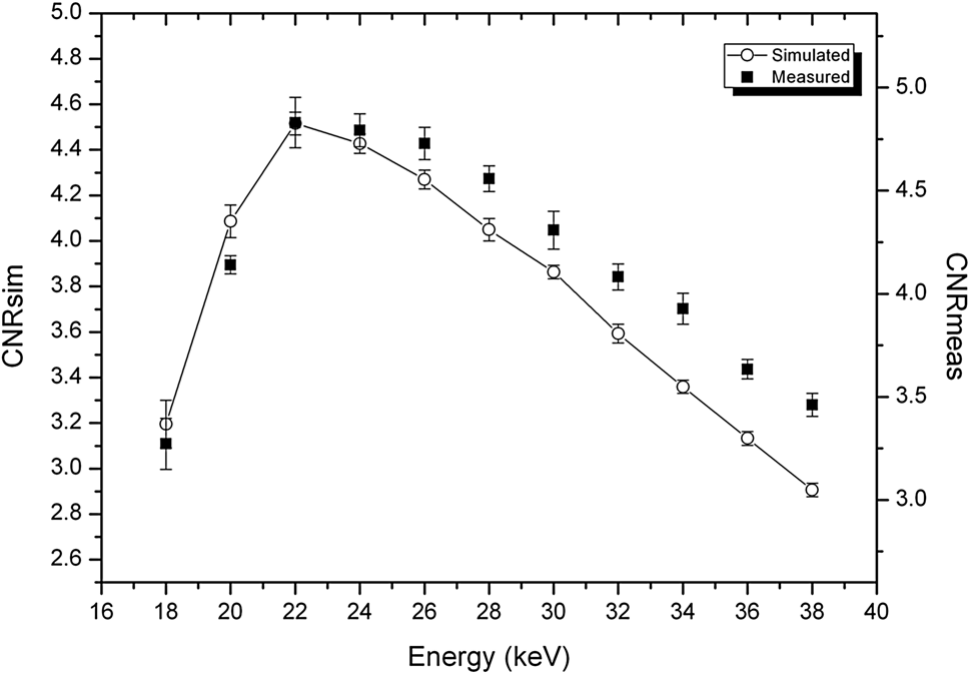
CNR as a function of energy for the T1 sample. Experimental data (solid square) and simulations (hollow circle).

In Fig. 5, the CNR for both the experimental and simulated data is reported for sample T1. The maximum of CNR is again at 22 keV, for both the simulation and experimental image. Also, the energy dependence of the two curves is very similar. The fact that the energy maximizing the CNR is rather low can be explained considering that the polyethylene attenuation coefficient, which composes most of the sample, is smaller than the one of adipose tissue.

Both surgical samples T2 and T3 have sizes resembling realistic breast dimensions, so the evaluation of CNR gives realistic information on which is the optimal energy in a clinical scenario. For these samples a potential additional source of discrepancy between the experimental and the simulated CNRs comes from the fact that the reconstructions of the actual samples have been segmented for the simulation. The arbitrary threshold employed to segment the gland and adipose tissues can slightly change the actual ratio between the glandular and the adipose volumes. This factor may influence the scale of the CNR and may induce some slight shift in the maximum of the simulated CNR.

**Figure 6:**
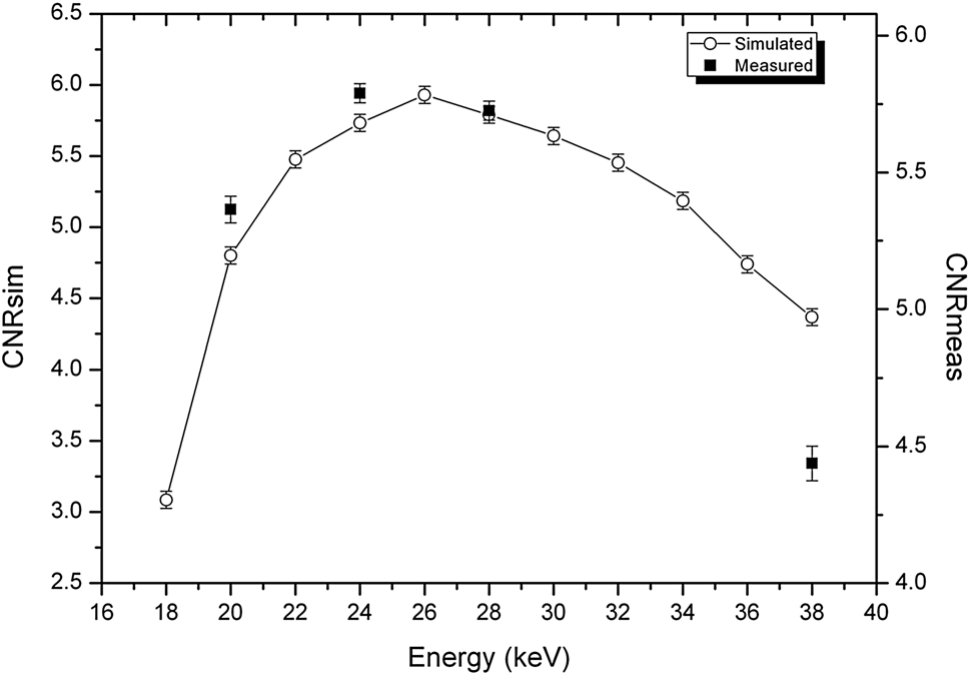
CNR for breast tissue T2, experimental (solid squares) and simulated (hollow circles).

In Fig. 6, the CNR for both the experimental and simulated data is reported for sample T2. For this sample the maximum of CNR on experimental data was found to be at 24 keV. It should be noted that, for this sample, the energy sampling is coarser (4 keV around 24 keV) hence the uncertainty on the maximum position is higher. The maximum of CNR for simulated data is found at 26 keV which is compatible with the experimental finding, considering the difference in the energy sampling. Higher energies, traditionally used in bCT^34,55^, cause a decrease of CNR, which becomes about 92% of its maximum value at 32 keV, about 80% at 36 keV and about 75% at 38 keV.

**Figure 7:**
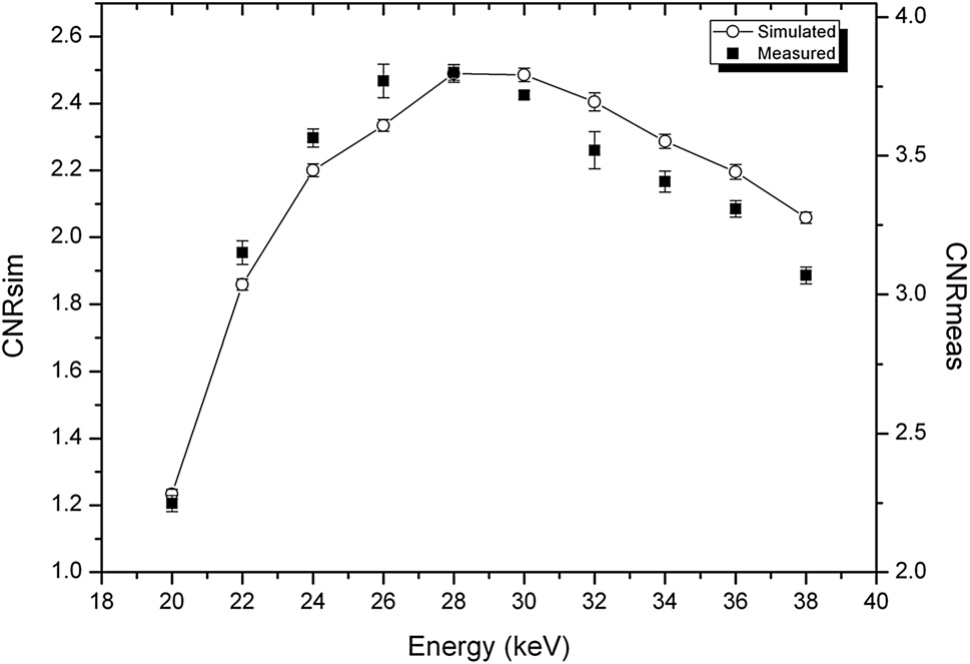
CNR for breast tissue T3, experimental (solid squares) and simulated (hollow circles).

In Fig. 7, the CNR for both the experimental and simulated data is reported for sample T3. For this sample, the maximum of CNR is at 28 keV, for both simulated and experimental data. However, even though the maxima on the sampled points are at the same energy, the actual peak for experimental CNR appears slightly shifted towards lower energies.

### Photon starvation

**Figure 8:**
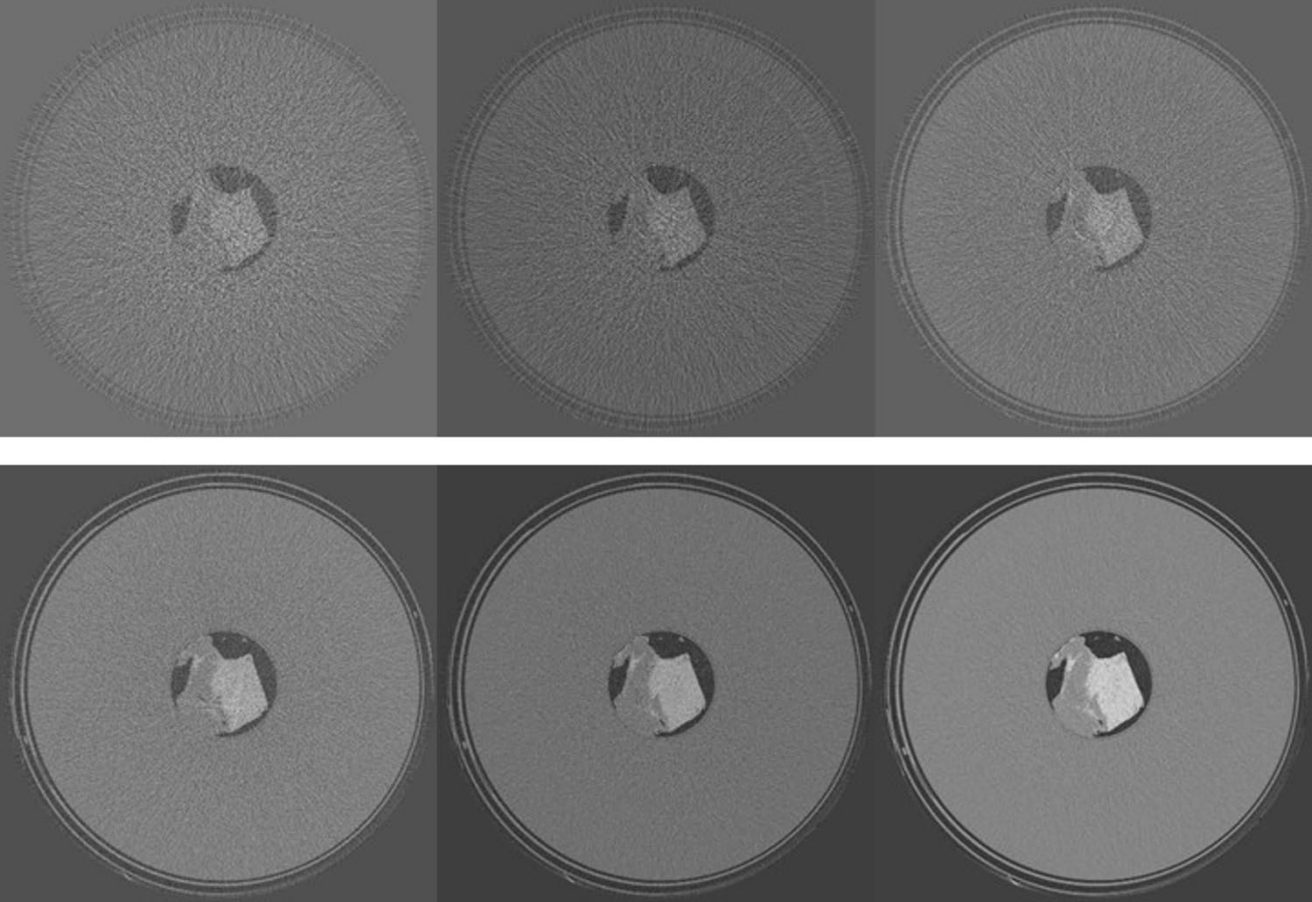
Reconstructed slices for breast sample T1 at the different mean glandular doses. Top, from left to right: 0.125, 0.25, 0.5 mGy. Bottom, from left to right: 2, 5 and 20 mGy.

For the T1 tissue sample, the dependence of CNR on MGD was evaluated at 22 keV. Images were acquired at 0.125, 0.25, 0.5, 1, 2, 5 and 20 mGy. CNR was evaluated always on the same ROIs, which were accurately selected in order to avoid ring artifacts (see Fig. 8), which would have altered the estimation of CNR. In Fig. S4 in the Supplementary Materials, the CNR (left panel) and the percentage of zero-counting pixels (right panel) are reported as a function of dose. The dose for each acquisition, the corresponding fluence rate in input to the sample in unit of (photons/mm^2^/projection), the measured *N*_*min*_, the percentage of zero-counting pixels and the CNR are reported in Table S1 (Supplementary Materials). CNR shows a behavior proportional to the square root of *N*_*min*_ down to 2 mGy (~ one count/pixel). For doses below 1 mGy, the CNR slightly increases with the decrease of *N*_*min*_. This effect is not realistic and it is due to the removal of the zero counting pixels. Actually, the preprocessing procedure induces an increasing smoothing in the processed images with the increase of the number of the removed zero counts, thus improving the CNR^44^.

In Fig. 8, the reconstructed slices on which CNR was calculated are shown, for different doses. At 20 mGy, the tissue is perfectly reconstructed, although a small ring artifact is visible close to the center of rotation. This artifact is due to a non-perfect equalization of two adjacent detector modules. At 5 mGy ring artifacts of the same nature, corresponding to the junctions of other detector modules, are visible, but the quality of the image is still very good. Below 2 mGy the ring artifacts start to significantly alter the image quality. Below 1 mGy, effects due to the photon starvation start to become dominant and image details are rapidly lost. This is also appreciable considering CNR in Fig. S4 and Table S1 (Supplementary Materials): below 1 mGy CNR is completely dominated by artifacts and no more represents actual contrast in the image.

**Figure 9:**
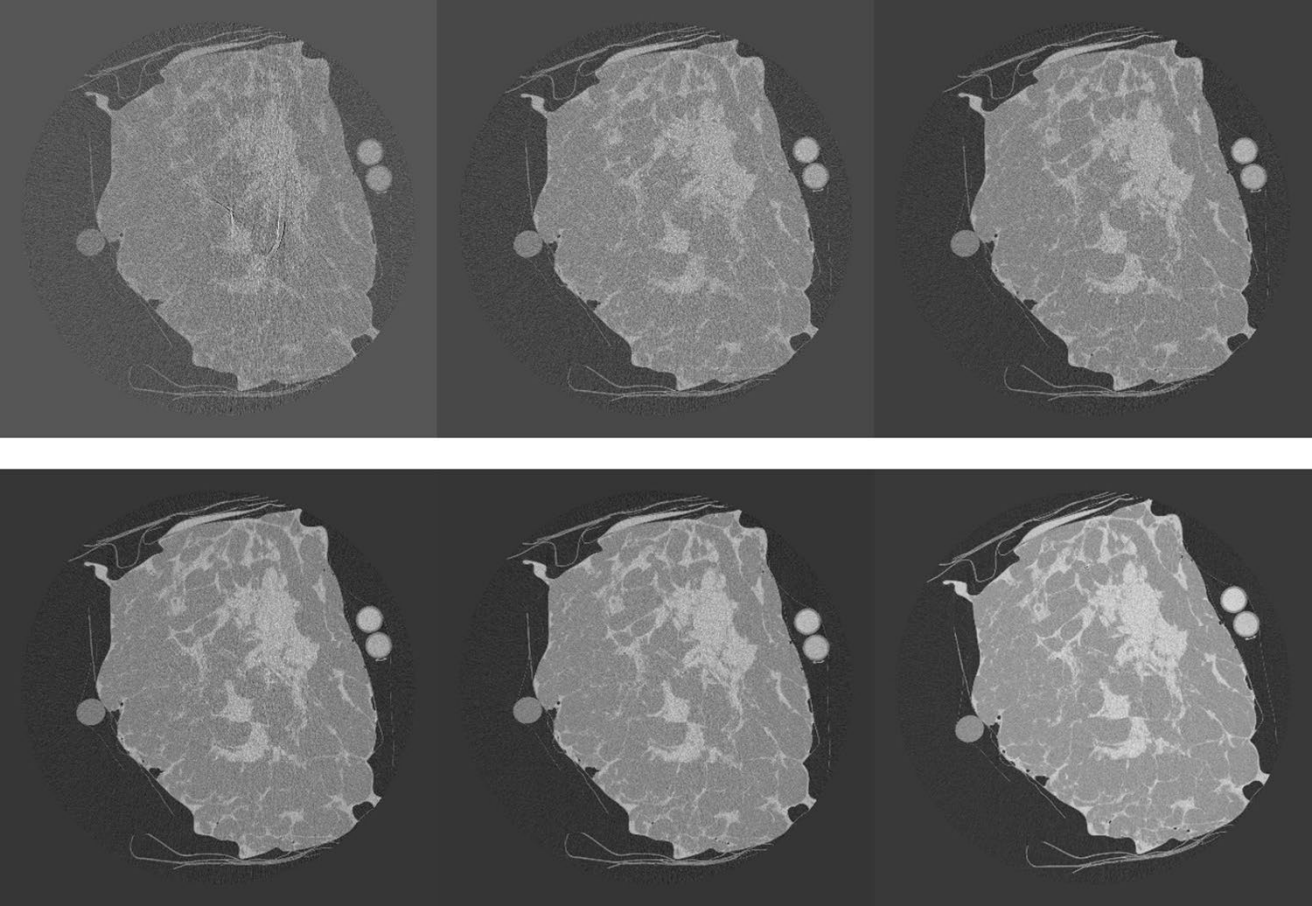
Reconstructed slices for breast sample T3 at the different mean glandular doses. Top, from left to right: 1, 2.5, 5 mGy. Bottom, from left to right: 7.5, 10 and 20 mGy.

For the T3 tissue sample, the dependence of CNR on MGD was evaluated at 28 keV. Images were acquired at 1, 2.5, 5, 7.5 10 and 20 mGy. The reconstructed slices on which CNR was calculated for the different doses are reported in Fig. 9.

In Fig. S5 in the Supplementary Materials, the CNR as a function of dose (left panel) and the percentage of zero-counting pixels as a function of dose (right panel) are reported. The dose for each acquisition, the corresponding fluence rate in input to the sample in unit of (photons/mm^2^/projection), the measured *N*_*min*_, the percentage of zero-counting pixels and the CNR are reported in Table S2 (Supplementary Materials).

For this phantom the used MGDs were higher. Hence, as expected, CNR shows a behavior proportional to the square root of the number of counts, also for the lowest evaluated doses. In these cases (in particular 2.5 and 1 mGy), the percentage of zero-counting pixels is quite relevant (25% and 56%, respectively).

**Figure 10:**
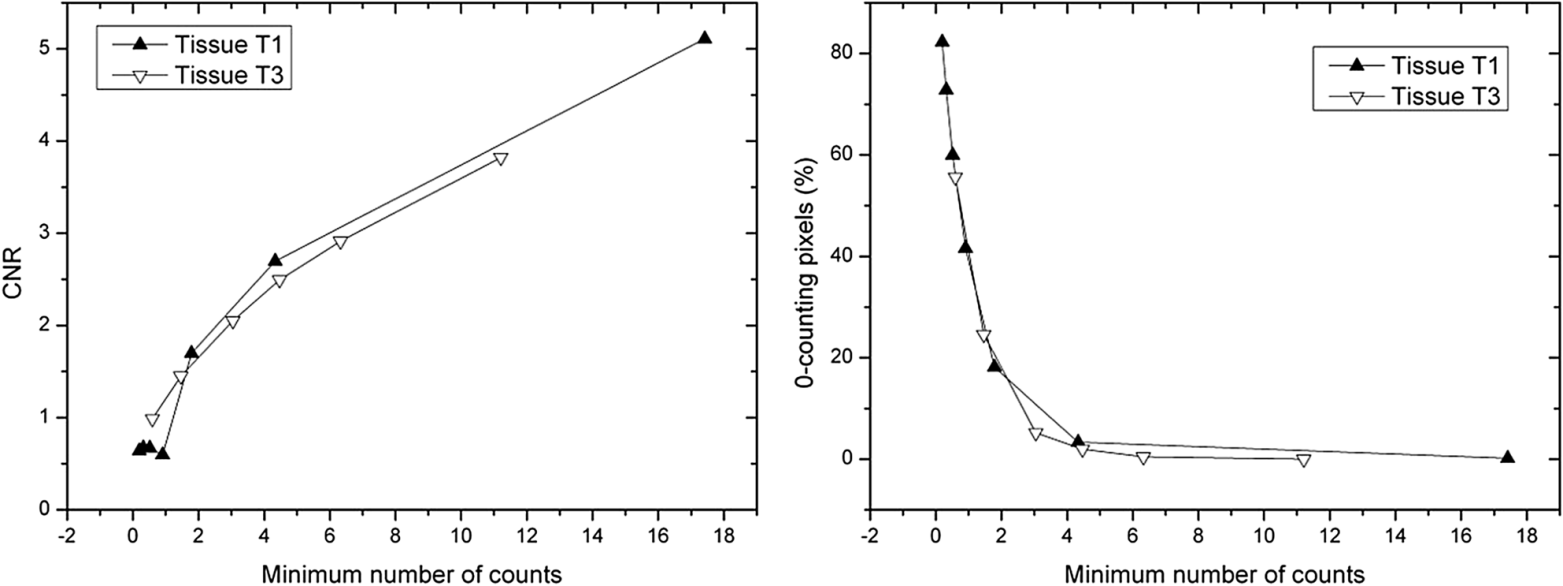
CNR (left) and percentage of zero-value pixels (right) for the two breast samples T1 (at 22 keV) and T3 (at 28 keV), expressed as a function of the minimum number of counts.

Since the actual parameter that influences the quality of the reconstructed images is the (minimum) number of counts in the projections, in Fig. 10 the CNR (left panel) and the percentage of zero-counting pixels (right panel) are expressed as a function of *N*_*min*_, for the two series of acquisitions. Despite the fact that the two CNR are calculated for two different samples, at two different energies and for different sets of doses, the curves overlap quite well, if the number of counts is still reasonable. As can be seen from Tables S1 and S2 (Supplementary Materials), this happens when the (average) number of counts is greater than 1 and the percentage of zero-counting pixels is below 25%.

## Conclusions

The experimental results presented in this work show that, for the actual breast specimens under investigation, the energy that maximizes the contrast-to-noise ratio between glandular and adipose tissue in phase retrieved computed tomography is around 26–28 keV. These results are in good agreement with the conclusions of (Delogu et al.^37^), who found, by using simulated cylindrical phantoms of different diameters and compositions, that 28 keV was an optimal choice for a large set of configurations. Our suggested energy interval is thus appreciably lower than the one predicted by previous studies, which simulated bCT in similar, though not identical, experimental conditions^34^. On the other hand, our findings better expand the conclusions of (Baran et al.^55^), who found their best results at 32 keV, as compared with 35 and 38 keV, being 32 keV the lowest investigated energy.

We have also shown that our simulation software is able to reproduce, to a large extent, the energy dependence of CNR for samples of known materials, for both absorption and phase-retrieved images. Concerning the observed discrepancies, these are mainly due to the different approach in dealing with zero-counting pixels or to the modeling of the detector (which is supposed to be ideal in the simulations).

In the case of breast specimens, another possible source of differences is that the actual composition of breast tissues is unknown. However, also with these uncertainties, the optimal energy is reasonably reproduced by the analytical simulation.

In conclusion, we found out that at least two photons per pixel per projection are necessary to maintain an acceptable image quality and to properly correct artifacts induced by photon starvation. This finding lays the bases for implementing procedures to be employed when aiming at performing low dose bCT. For example, when the condition of two photons per pixel is not met, the increase of the exposure time for each projection (i.e. the reduction of projections) can be considered. However, when performing a CT scan in continuous acquisition, this approach can induce motion artifacts along the tangential profile^27^. Alternatively, a re-binning of pixels (2 × 2) can be implemented at the cost of a reduced spatial resolution. Another potential hardware solution could be the adoption of a bow tie filtration system allowing a more homogeneous photon distribution at the detector. Anyhow, this would require the adoption of many different filters, each optimized for a specific breast size and energy, which have to be replaced for each patient, thus potentially being unpractical in the clinical routine.

## Supplementary information

Supplementary file1
