# Supplementary material for: Experimental optimization of the energy for breast-CT with synchrotron radiation
Source: Sci Rep. 2020 Oct 15;10:17430. doi: 10.1038/s41598-020-74607-7 (PMC7567093; doi:10.1038/s41598-020-74607-7)
Supplement: Supplementary file 1 — Supplementary file1 [file 41598_2020_74607_MOESM1_ESM.docx]

Experimental optimization of the energy for Breast-CT with synchrotron radiation

**Piernicola Oliva^1,2^, Vittorio Di Trapani^3,4,*^,** **Fulvia Arfelli^5,6^, Luca Brombal^5,6^ , Sandro Donato^7,8,9^, Bruno Golosio^10,2^, Renata Longo ^5,6^, Giovanni Mettivier^11,12^, Luigi Rigon^5,6^, Angelo Taibi^13,14^, Giuliana Tromba^9^, Fabrizio Zanconati^15^, Pasquale Delogu^3,4^**

^1^ Dipartimento di Chimica e Farmacia, Università di Sassari, Sassari, Italy.

^2^ I.N.F.N. Sezione di Cagliari, Cagliari, Italy.

^3^ Dipartimento di Scienze Fisiche, della Terra e dell’Ambiente, Università di Siena, Siena, Italy.

^4^ I.N.F.N. Sezione di Pisa, Pisa, Italy.

^5^ Dipartimento di Fisica, Università di Trieste, Trieste, Italy.

^6^ I.N.F.N. Sezione di Trieste, Trieste, Italy.

^7^ Dipartimento di Fisica, Università della Calabria, Cosenza, Italy.

^8^ I.N.F.N. Laboratori Nazionali di Frascati, Frascati, Italy.

^9^ Elettra-Sincrotrone Trieste SCpA, Basovizza, Italy.

^10^ Dipartimento di Fisica, Università di Cagliari, Cagliari, Italy.

^11^ Dipartimento di Fisica, Università di Napoli Federico II, Napoli, Italy.

^12^ I.N.F.N. Sezione di Napoli, Napoli, Italy.

^13^ Dipartimento di Fisica e Scienze dalla Terra, Università di Ferrara, Ferrara, Italy.

^14^ I.N.F.N. Sezione di Ferrara, Ferrara, Italy.

^15^ [Dipartimento di Scienze Mediche Chirurgiche e della Salute](https://dsm.units.it/it), Università di Trieste, Trieste, Italy.

* vittorio.ditrapani@pi.infn.it

# Supplementary Materials


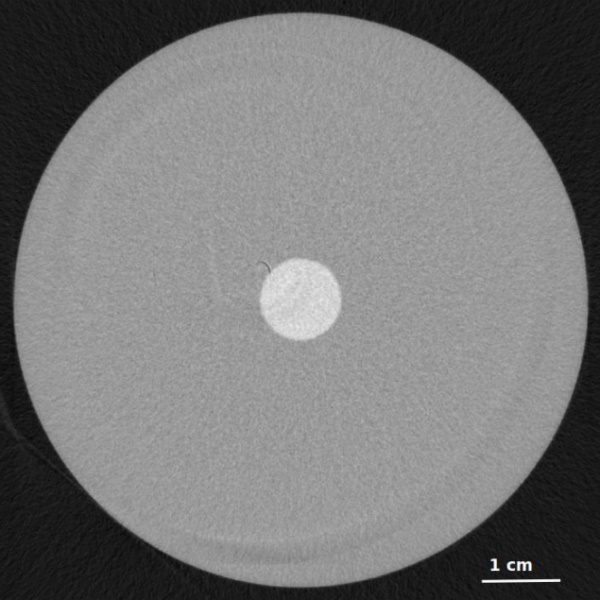

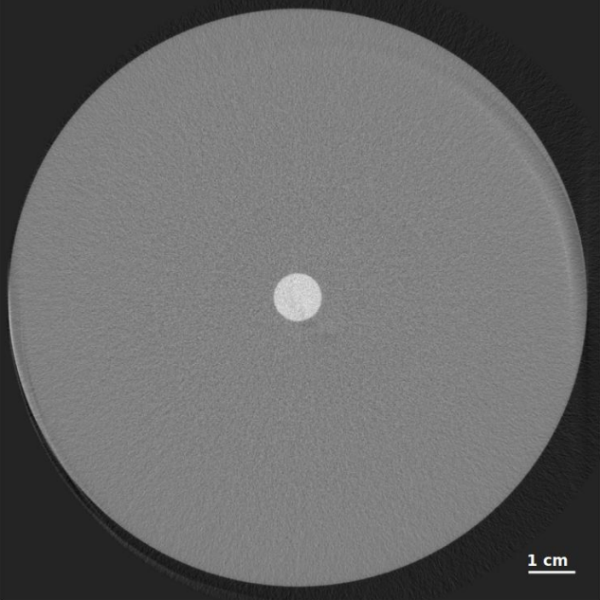


**Figure S1.**  Tomographic reconstruction of the phantoms. Left: P1. Right: P2. The diameter of the central detail is 1cm, while the diameter of the external cylinder is 7 cm for P1 and 12 cm for P2.

**
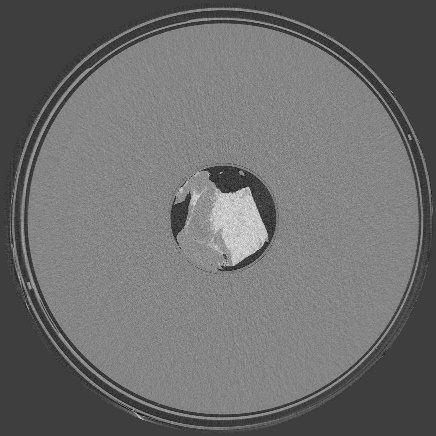

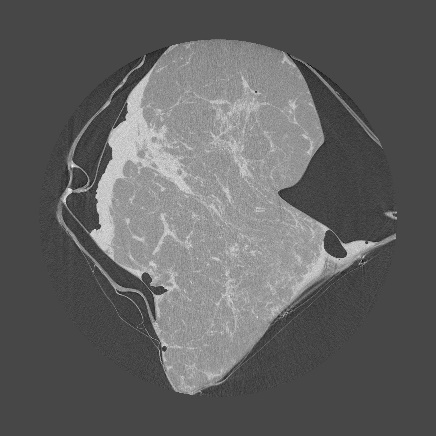

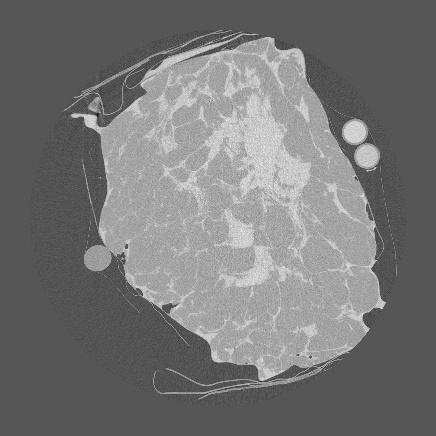
**

**Figure S2.**  Slices of the breast samples. Left: T1. Center: T2. Right: T3


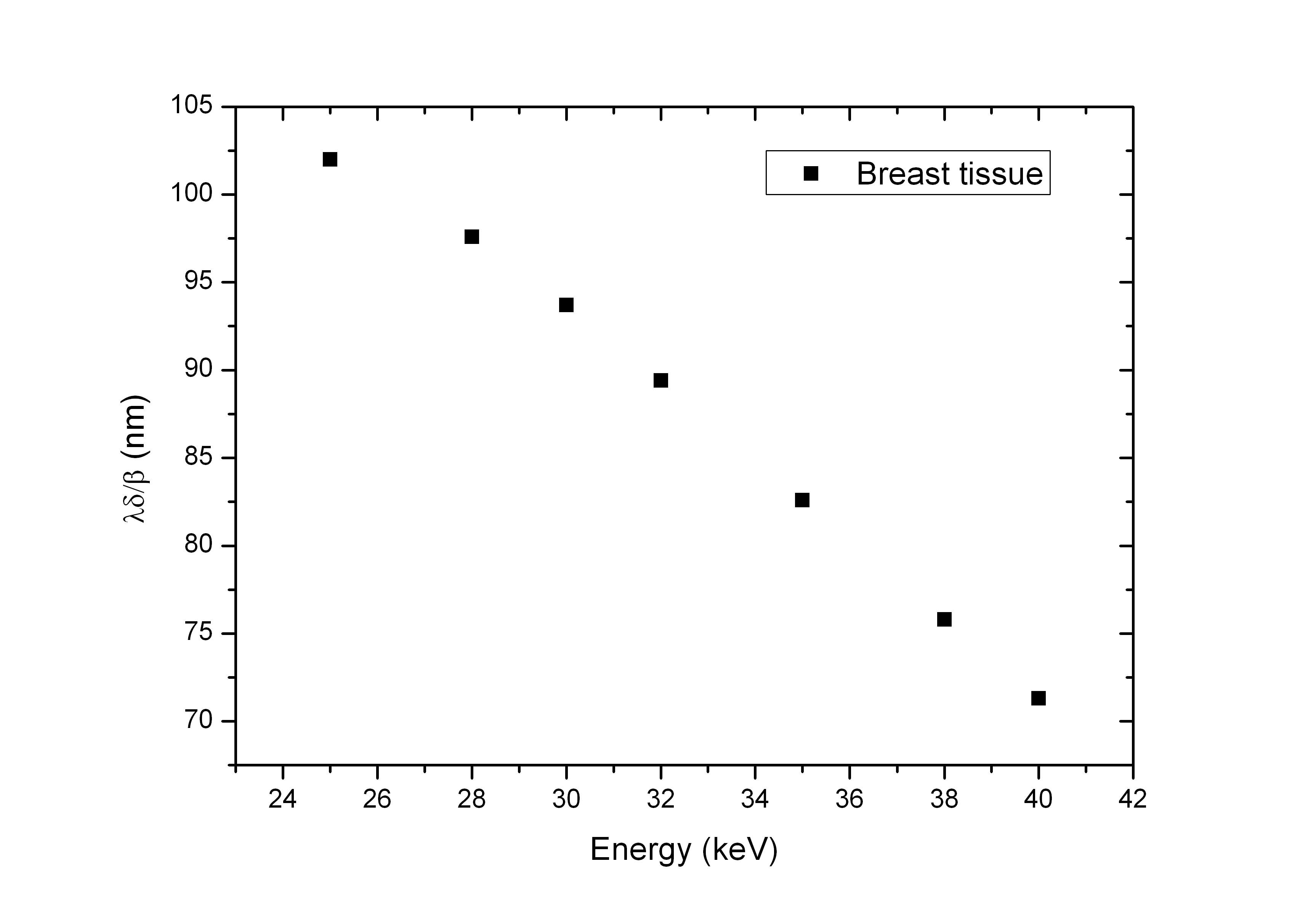


**Figure S3.** $\lambda\delta/ \beta$ is plotted for breast tissue, as a function of energy. Data from University of Melbourne 1.

**
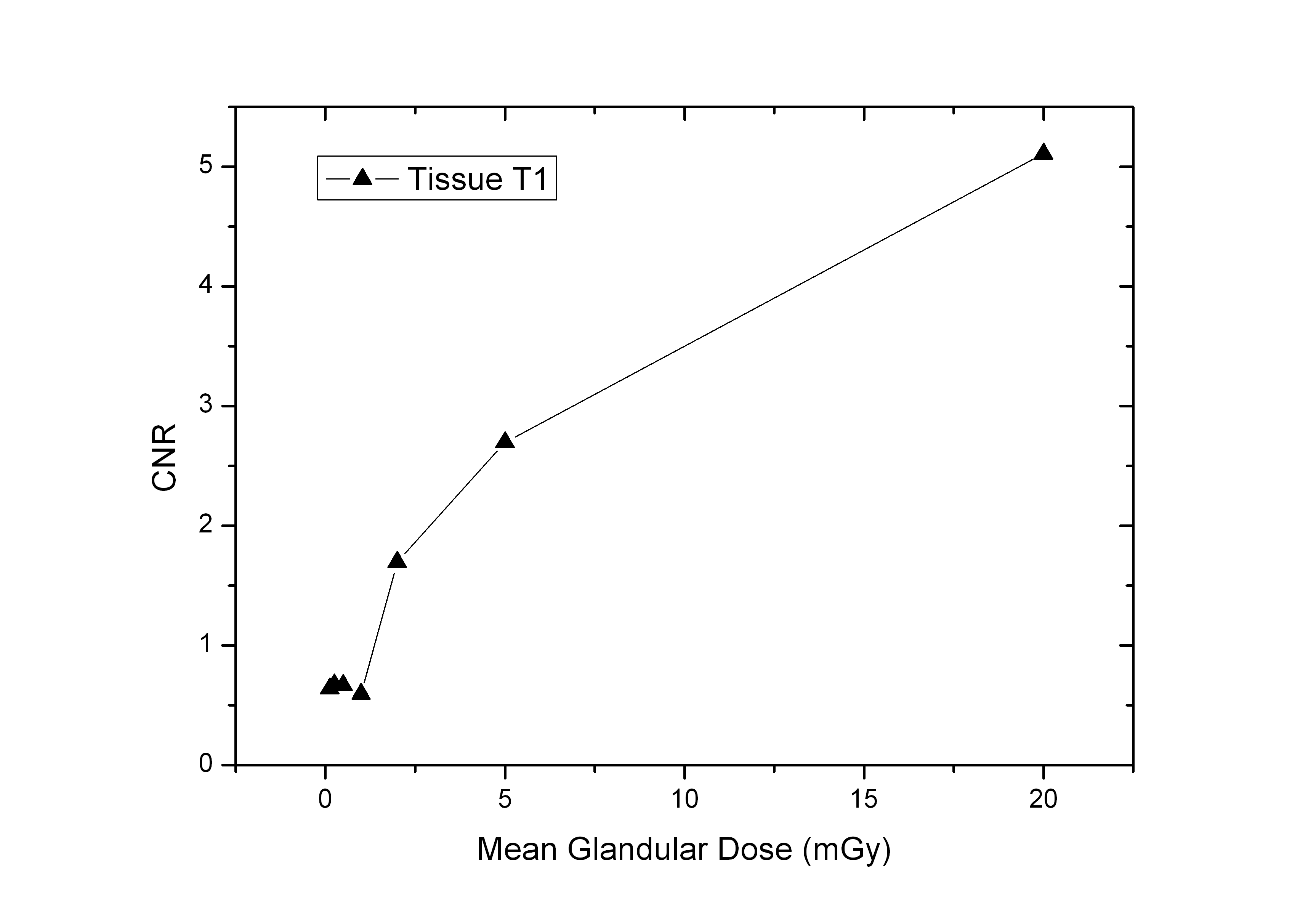

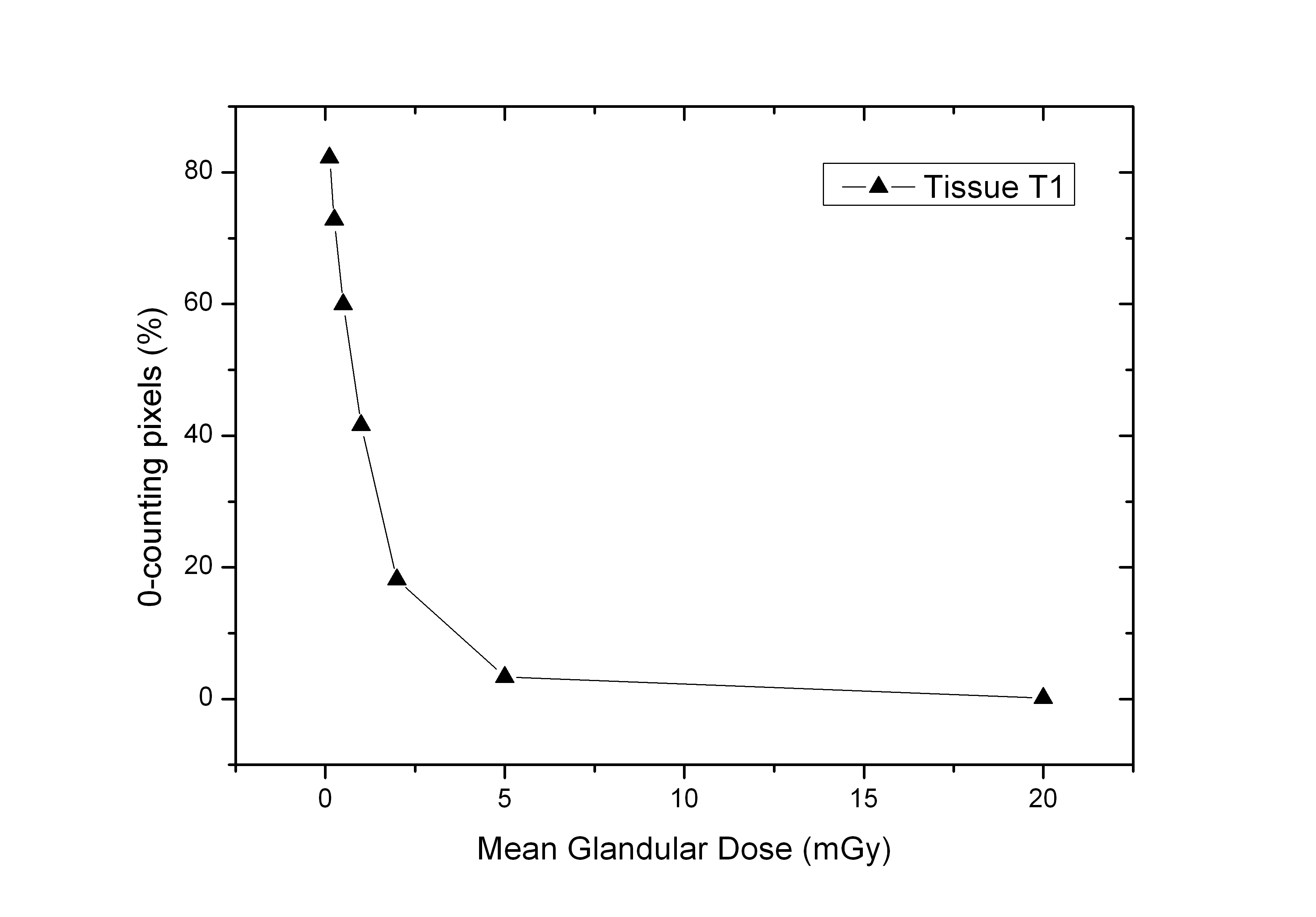
**

**Figure S4.** CNR (left) and percentage of zero-value pixels (right) as a function of mean glandular dose for breast sample T1.


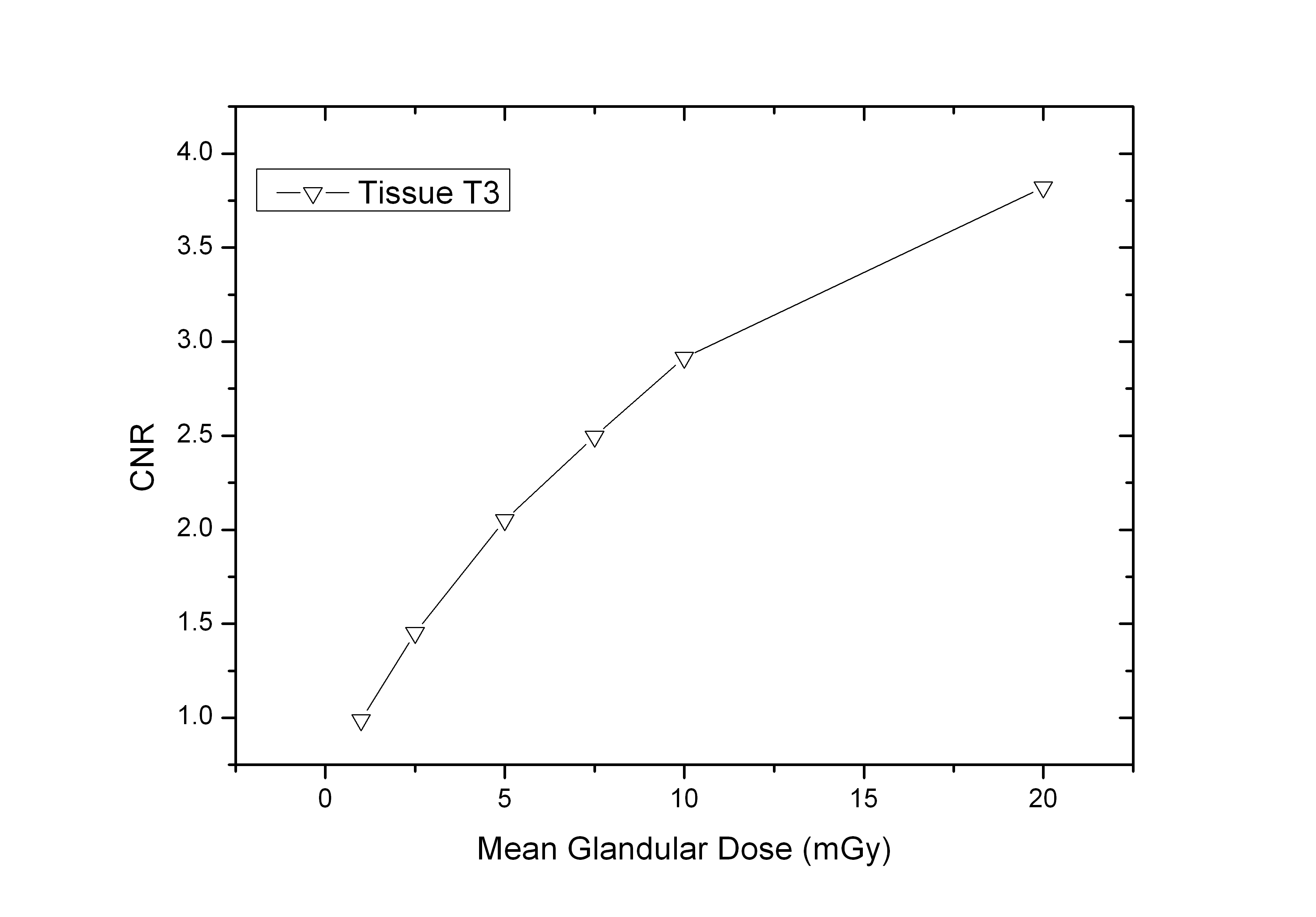

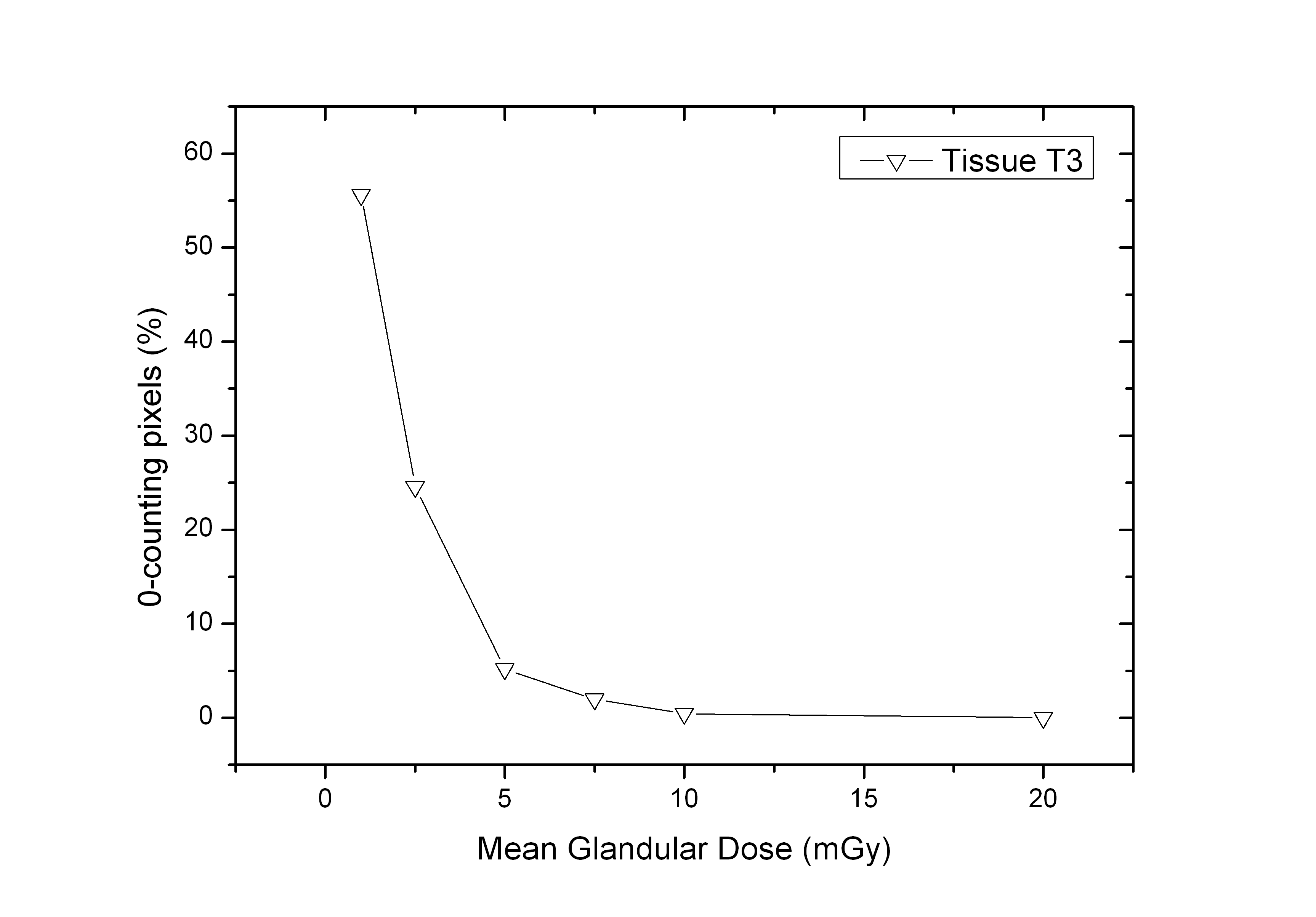


**Figure S5.** CNR (left) and percentage of zero-value pixels (right) as a function of mean glandular dose for breast sample T3

| **Mean Glandular Dose (mGy)** | **Fluence rate (photons/mm^2^/projection)** | **N_min_** | **0-counting pixels (%)** | **CNR** |
| --- | --- | --- | --- | --- |
| 0.12 | $2.1\cdot10^3$ | 0.2 | 82 | 0.64 |
| 0.25 | $4.1\cdot10^3$ | 0.3 | 73 | 0.67 |
| 0.5 | $8.3\cdot10^3$ | 0.5 | 60 | 0.68 |
| 1 | $1.7\cdot10^4$ | 0.9 | 42 | 0.6 |
| 2 | $3.2\cdot10^4$ | 1.8 | 18 | 1.7 |
| 5 | $8.3\cdot10^4$ | 4.3 | 3.3 | 2.7 |
| 20 | $3.5\cdot10^5$ | 17.4 | 0.14 | 5.1 |

**Table S1.** For the breast sample T1, the fluence rate at the sample position, the minimum for each set of projections of the average number of counts in a central area of the image (N_min_), the percentage of zero-counting pixel and the Contrast-to-noise ratio are reported for the different mean glandular doses.

| **Mean Glandular Dose (mGy)** | **Fluence rate (photons/mm^2^/projection)** | **N_min_** | **0-counting pixels (%)** | **CNR** |
| --- | --- | --- | --- | --- |
| 1 | $1.8\cdot10^4$ | 0.6 | 56 | 1 |
| 2.5 | $4.5\cdot10^4$ | 1.5 | 25 | 1.45 |
| 5 | $9.9\cdot10^4$ | 3.1 | 5 | 2.05 |
| 7.5 | $1.4\cdot10^5$ | 4.5 | 2 | 2.5 |
| 10 | $2.0\cdot10^5$ | 6.3 | 0.43 | 2.9 |
| 20 | $3.9\cdot10^5$ | 11.2 | 0 | 3.8 |

**Table S2.** For the breast sample T3, the fluence rate at the sample position, the minimum for each set of projections of the average number of counts in a central area of the image (N_min_), the percentage of zero-counting pixel and the Contrast-to-noise ratio are reported for the different mean glandular doses.

# References

x

| 1. | Taylor, J. A., CSIRO TS imaging, Available at <http://ts-imaging.science.unimelb.edu.au//Services//Simple//ICUtilXdata.aspx> (2015). |
| --- | --- |

x
